# Myelin Breakdown in Human Huntington’s Disease: Multi-Modal Evidence from Diffusion MRI and Quantitative Magnetization Transfer

**DOI:** 10.1016/j.neuroscience.2017.05.042

**Published:** 2019-04-01

**Authors:** José Bourbon-Teles, Sonya Bells, Derek K. Jones, Elizabeth Coulthard, Anne Rosser, Claudia Metzler-Baddeley

**Affiliations:** a Cardiff University Brain Research Imaging Centre (CUBRIC), School of Psychology, and Neuroscience and Mental Health Research Institute, Cardiff University, UK; b School of Psychology, Faculty of Health Sciences, Australian Catholic University, Victoria, Australia; c Clinical Neurosciences, Bristol University, UK; d Brain Repair Group, School of Biosciences, Cardiff University, UK

**Keywords:** AD, axial diffusivity, ATR, anterior thalamic radiation, BG, basal ganglia, CC, corpus callosum, CST, cortico-spinal tract, dRL, damped Richardson-Lucy algorithm, DSST, Digit Symbol Substitution Test, FA, fractional anisotropy, fODFs, fiber orientation density functions, FWE, Free Water Elimination, HARDI, high angular resolution diffusion imaging, HD, Huntington’s disease, MMPF, macromolecular proton fraction, MoCA, Montreal Cognitive Assessment, PCA, principal component analysis, PFC, prefrontal cortex, qMT, quantitative magnetization transfer, RD, radial diffusivity, ROI, region of interest, SMA, supplementary motor area, SPGR, spoiled gradient recalled-echo, WM, white matter, YoE, years of education, Huntington’s disease, myelin, white matter, basal ganglia, cognition, clinical markers

## Abstract

Huntington’s disease (HD) leads to white matter (WM) degeneration that may be due to an early breakdown in axon myelination but *in vivo* imaging correlates of demyelination remain relatively unexplored in HD compared to other neurodegenerative diseases. This study investigated HD-related effects on a putative marker of myelin, the macromolecular proton fraction (MMPF) from quantitative magnetization transfer and on fractional anisotropy, axial and radial diffusivity from diffusion tensor MR-imaging. Microstructural differences were studied in WM pathways of the basal ganglia and motor systems known to be impaired in HD: the corpus callosum, the cortico-spinal tract, the anterior thalamic radiation, fibers between prefrontal cortex and caudate and between supplementary motor area and putamen. Principal component analysis was employed for dimensionality reduction. Patients showed reductions in a component with high loadings on MMPF in all WM pathways and a trend for increases in a component loading on axial and radial diffusivities but no differences in a component loading on fractional anisotropy. While patients’ performance in executive functioning was impaired, their working memory span was preserved. Inter-individual differences in the diffusivity component correlated with patients’ performance in clinical measures of the United Huntington Disease Rating Scale. In summary, HD-related reductions in MMPF suggest that myelin breakdown contributes to WM impairment in human HD and emphasize the potential of quantitative MRI metrics to inform about disease pathogenesis. Disease severity in manifest HD, however, was best captured by non-specific diffusivity metrics sensitive to multiple disease and age-related changes.

## Highlights

• The macromolecular proton fraction (MMPF), an MRI marker of myelin, was reduced in Huntington’s disease (HD).
• MMPF reductions in white matter suggest myelin breakdown.
• HD was associated with reductions in basal ganglia volume.
• HD was associated with poor executive functioning but preserved working memory capacity.
• Axial and radial diffusivities as unspecific metrics of white matter changes correlated with clinical markers of disease.

## Introduction

Huntington’s disease (HD) is a progressive neurodegenerative disease caused by an expansion and instability of the cytosine-adenine-guanine triplet repeat (CAG repeat) in the Huntingtin gene. Individuals with HD exhibit a progressive decline of motor and cognitive functions, notably executive functions (Mörkl et al., 2016), as well as disturbances in affect and motivation (Papoutsi et al., 2014).

Mutation carriers not only show gray matter atrophy in the basal ganglia (BG) but also subtle and progressive white matter (WM) impairment many years before the onset of any clinical symptoms (Dayalu and Albin, 2015). Microstructural impairments at prodromal disease stages have been identified for major WM bundles including the corpus callosum, the anterior thalamic radiation and the cortico-spinal tract (Matsui et al., 2015, Novak et al., 2014, Novak et al., 2015, Phillips et al., 2014, Phillips et al., 2015, Odish et al., 2015, Poudel et al., 2015, Steventon et al., 2015). Alterations in WM microstructure are related to early striatal atrophy (Novak et al., 2014), to disease progression (Rosas et al., 2006, Gregory et al., 2015) and early cognitive dysfunctions (Matsui et al., 2015).

The majority of studies into WM in HD (Rosas et al., 2006, Gregory et al., 2015, Poudel et al., 2015) have employed diffusion tensor magnetic resonance imaging (DT-MRI) to characterize and quantify WM microstructural changes with metrics of fractional anisotropy (FA) or diffusivities (Basser et al., 1994). DT-MRI indices are sensitive to microstructural changes in many neurodegenerative diseases including Mild Cognitive Impairment (Metzler-Baddeley et al., 2012a; Metzler-Baddeley et al., 2012b), Alzheimer’s disease (Acosta-Cabronero and Nestor, 2014), Parkinson’s Disease (Bohnen and Albin, 2011, Mole et al., 2016) and Multiple Sclerosis (Abhinav et al., 2014). However, changes in DT-MRI metrics are difficult to interpret biologically since they lack any specific pathological counterpart and can occur due to changes in the fiber organization and orientation complexity as well as in axon caliber and myelination (Beaulieu and Allen, 1994, De Santis et al., 2014). By using a multi-modal approach that complements DT-MRI metrics with other modalities sensitive to WM tissue properties one may therefore gain more specific clues about the potential underlying mechanisms affecting WM microstructure in HD.

Bartzokis et al. (2007) utilized MRI at two different field strengths (0.5 T and 1.5 T) to quantify the iron content of ferritin molecules in white and gray matter of eleven HD patients compared to controls. In patients, decreased ferritin iron levels were found in the genu of the corpus callosum and the frontal WM accompanied by increased levels in the BG while no differences between the groups were present in the hippocampus and the thalamus as control regions. More recently, Phillips et al. (2015) using T_2_^∗^-weighted imaging also reported elevated iron levels in the cortico-spinal tract of HD patients. As iron is required for the formation of myelin, which in turn is essential for the development and maintenance of healthy brain function, these findings suggest myelin disturbance (Fields, 2014). Myelin insulates and protects axons and enables saltatory conduction, i.e., the process of action potentials moving in discrete jumps along a myelinated axon to speed up information transfer between neurons. In the central nervous system myelin is produced and repaired by oligodendrocyte glia cells, which are the cells with the highest iron concentration in the human brain (Koeppen, 1995).

The view that myelin disturbance may play a critical role in HD is backed up by recent evidence from post-mortem and animal studies. Using a variety of techniques, including carbon dating, one *post-mortem* study of brain tissue from individuals with HD has suggested decreased proliferation of striatal oligodendrocyte lineage cells (Ernst et al., 2014). Furthermore, Teo et al. (2016) reported thinner myelin sheaths in fibers of the corpus callosum in HD knock-in mice at an early age before any striatal neuronal loss could be detected. Similarly, reduced levels of myelin regulatory factor and myelin basic protein and decreased numbers of myelinated axons in the corpus callosum were reported in other HD mice model studies (Huang et al., 2015, Jin et al., 2015). Together these results suggest that an early breakdown of myelination followed by homeostatic responses of increased levels of oligodendrocytes and iron may contribute to the pathogenesis of HD (Bartzokis et al., 2007). However, *in vivo* correlates of demyelination in human HD remain relatively unexplored compared to other neurodegenerative diseases such as multiple sclerosis, despite their potential in helping to better understand the pathogenesis of the disease and in providing biomarkers for the efficacy of therapeutics.

The present study investigated HD-related differences in WM microstructure by utilizing both high angular resolution diffusion imaging (HARDI) (Tuch et al., 2002) and quantitative magnetization transfer imaging (qMT) (Henkelman et al., 1993, Henkelman et al., 2001) which provides improved myelin specificity compared to diffusion MRI. qMT estimates the liquid and semisolid constituents of tissue by applying an off-resonance radiofrequency pulse with time-varying amplitude to selectively saturate the macromolecular magnetization. This results in a reduction of the measured signal due to the magnetization transfer between saturated macromolecules and free water (Sled and Pike, 2000, Ramani et al., 2002). Molecules associated with myelin have been shown to dominate the magnetization transfer exchange process in WM (Koenig, 1991, Odrobina et al., 2005) and the relative density of the macromolecular pool, the macromolecular proton fraction (MMPF), has been found to be specific to myelin content of the optic nerve in shiverer *versus* control mice (Ou et al., 2009), to be sensitive to demyelination processes in multiple sclerosis (Levesque et al., 2010, Liu et al., 2015) and to depend on the myelin content of WM in *post-mortem* histology studies of human multiple sclerosis brains (Schmierer et al., 2007). Thus, although magnetization transfer will also be influenced by other processes such as inflammation and T_1_ changes, MMPF has been proposed as a proxy MRI marker of myelin (Serres et al., 2009b, Serres et al., 2009a).

The aim of the present study was to investigate whether MMPF as a marker of myelin would be a sensitive MRI metric for WM microstructural alterations in HD. For the purpose of comparability with previous research (Gregory et al., 2015, Poudel et al., 2015) we also studied HD-related changes in FA, radial diffusivity (RD) and axial diffusivity (AD) from DT-MRI, appreciating however that there are inherent problems of interpreting differences in these metrics in terms of myelin breakdown or axonal degeneration (Wheeler-Kingshott and Cercignani, 2009).

**Fig. 1:**
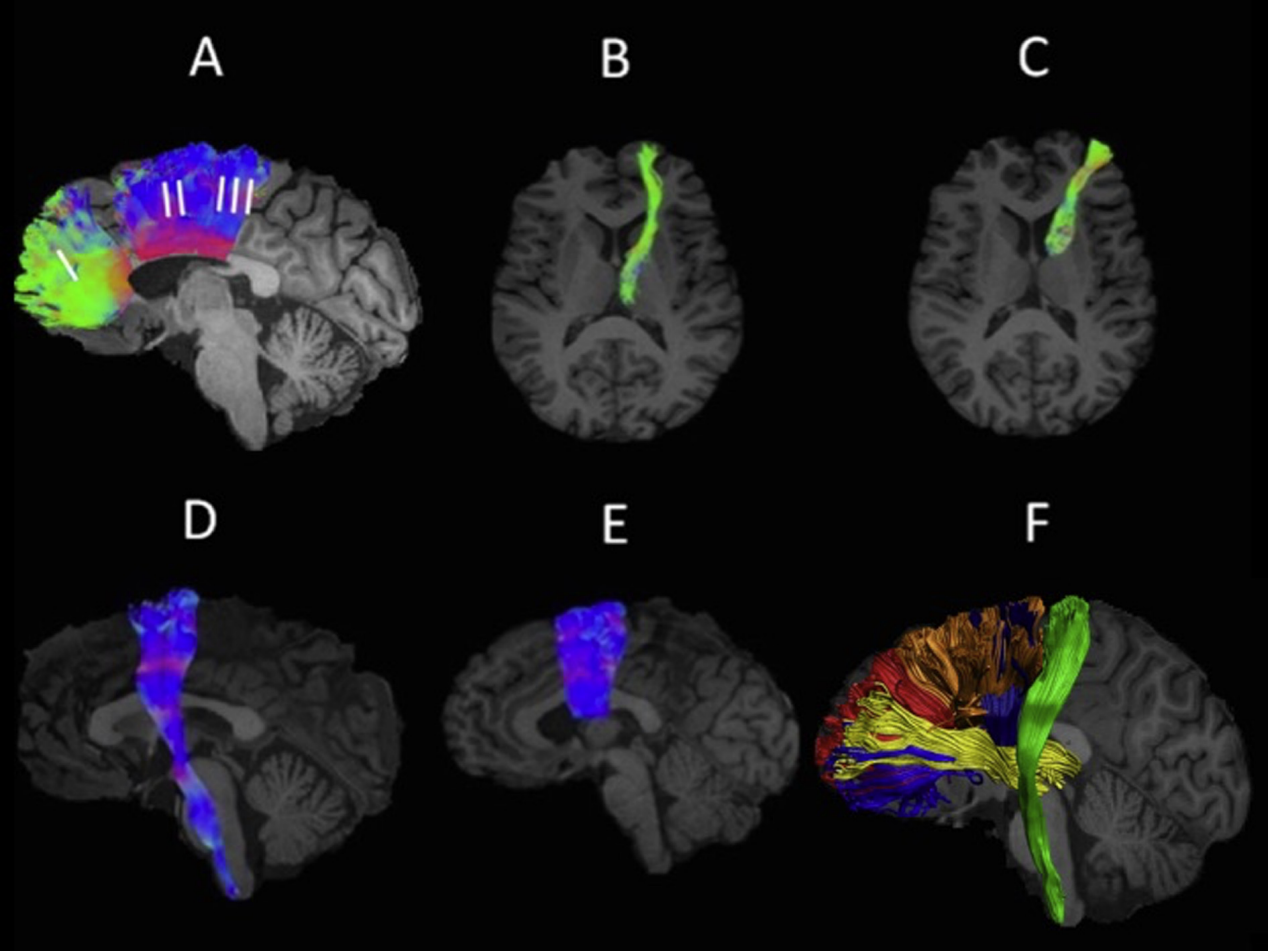
White matter pathway regions of interest. Sagittal and axial views of the reconstructed white matter pathways displayed on a T_1_-weighted image for one control participant. (A) segments I, II, and III of the corpus callosum (Hofer and Frahm, 2006), (B) anterior thalamic radiation, (C) prefrontal cortex – caudate pathway, (D) cortico-spinal tract and (E) supplementary motor area – putamen pathway. Fiber directions are color-coded with green indicating directions along the coronal, blue along the axial and red along the sagittal plane (Pajevic and Pierpaoli, 1999). (F) displays all reconstructed pathways on a sagittal view of the left hemisphere for another control participant. The corpus callosum segments are displayed in red (segment I) and orange (segments II and III), the anterior thalamic radiation in yellow, the prefrontal cortex – caudate fibers in blue, the supplementary motor area – putamen fibers in dark blue and the cortico-spinal tract in green.

WM microstructure was studied in pathways of the BG and motor systems which are known to be affected in HD, i.e., the anterior thalamic radiation (Matsui et al., 2015), the cortico-spinal tract (Phillips et al., 2015) and distinct sections of the corpus callosum (Hofer and Frahm, 2006) (Fig. 1). In addition, we reconstructed connections that form part of the BG loops, i.e., pathways between the prefrontal cortex (PFC) and the caudate nucleus (PFC-caudate) and between the supplementary motor area (SMA) and the putamen (SMA-putamen) (Alexander and Crutcher, 1990, Postuma and Dagher, 2006) (Fig. 1). Average indices of MMPF, FA, AD and RD were obtained for each of these tracts.

Following previous evidence suggesting impaired myelination as a significant contributor to WM abnormalities in HD (Bartzokis et al., 2007, Ernst et al., 2014, Teo et al., 2016) our primary hypothesis predicted lower MMPF in the WM pathways of patients compared to controls. Based on previous evidence (e.g. Gregory et al., 2015) we also expected reduced FA and higher RD and AD in patients relative to controls.

As secondary research questions we investigated the potential relationships between variation in WM microstructure and variation in gray matter volume of the bilateral BG (caudate, the putamen, the globus pallidus) and the thalamus. We also explored correlations between inter-individual variations in WM microstructure and cognitive functions as well as clinical markers from the United Huntington’s Disease Rating Scale (UHDRS) (Siesling et al., 1998) to assess brain-function relationships.

## Experimental procedures

### Participants

This study obtained National Health Service (NHS) Research Ethics Committee approval (13/WA/0326) and all participants gave written informed consent in accordance with the Declaration of Helsinki. Twenty-four patients with manifest HD and one pre-symptomatic individual (total *n* = 25) were recruited from the South Wales HD clinic based in Cardiff and the Bristol HD clinic. A group of age- and sex-matched healthy controls (*n* = 13) were recruited from the Cardiff University School of Psychology community panel and from patients’ spouses, carers or family members. Table 1 summarizes the patients’ demographic and some background clinical characteristics i.e., their CAG repeat length, their UHDRS-Total Motor Score (TMS) and UHDRS-Functional Assessment Score (FAS) and information about their medication.

**Table 1:** Patients’ demographics and background clinical information

Patients	Age	Sex	Length of CAG repeats	TMS	FAS	Medication
HD01	42	F	45	41	21	Olanzepine 5 mg, Citalopram 20 mg, Pregabalin 50 mg, Depakote 250 mg
HD02	46	F	40	9	24	Nil
HD03	24	M	43	2	25	Nil
HD04	38	F	45	10	22	Amitriptyline 75 mg, Omeprazole 10 mg, Prochlorperazine 10 mg,
HD05	60	M	43	48	17	Olanzepine 12 mg
HD06	45	F	42	46	16	Natriliz 1.5 mg, Rosuvasatin 10 mg, Atenolol 25 mg Carioplan 5 mg, Quetiapine 700 mg Metformin 500 mg, Indomethocin 25 mg, Pendopril 4 mg
HD07	55	M	43	82	13	Olanzapine 20 mg, Propanolol 30 mg, Cetrizine, Fortisips, Temazepam 10 mg
HD08	49	M	42	7	23	Nil
HD09	64	M	41	19	23	Citalopram 40 mg
HD10	44	F	47	72	1	Trazodone 300 mg, Abilify 5 mg, Temazepam 10 mg, Pregabalin 150 mg.
HD11	44	F	46	35	24	Citalopram 40 mg, Zopiclone 37.5 mg, Co-codamol 2 tables
HD12	64	M	40	35	22	Aspirin 75 mg, Atorvastatin 40 mg, Perindopril 4 mg, Sumatriptan 50 mg
HD13	46	M	45	35	13	Gabapentin 600 mg, Citalopram 30 mg,
HD14	22	M	51	17	23	Citalopram 30 mg
HD15	28	F	51	61	18	Perampanel 8 mg, Lamotrigine 100 mg, Levetiracetam 1500 mg, Clobazam up to 10 mg
HD16	47	M	46	69	18	Sertraline 50 mg,
HD17	62	F	41	4.	25	Novate ointments, Naproxen
HD18	50	M	40	0	25	Nil
HD19	68	F	43	40	17	Mirtazapine 30 mg
HD20	58	M	43	0	25	Atorvastatin 20 mg
HD21	58	M	41	37	25	Amlodipine 5 mg, Bendroflumethiazide 2.5 mg, Metformin 500 mg, Ramipril 2.5 mg, Sertraline 100 mg
HD22	30	F	42	0	25	Nil
HD23	37	F	46	6	25	Co-codamol 30/500, Omeprazole 10 mg, Ventolin inhaler 2 puffs, Amitriptyline 50 mg
HD24	51	M	43	20	23	Co-codamol 500 mg, Brufen 400 mg
HD25	47	F	41	5	25	Kliofem 2 mg, Amitriptyline 150 mg, Omeprazole 20 mg, Rizatriptan 10 mg, Loratidine 10 mg
Mean	47.2	–	44	28	20.7	
SD	12.5		3.1	24.9	5.65	

*Abbreviations:* CAG = cytosine-adenine-guanine, F = Female, M = Male, TMS = Total Motor Score out of 124 (the higher the scores the more impaired the performance). FAS = Functional Assessment Score out of 25 (the higher the scores the better the performance). HD = Huntington’s disease, SD = Standard Deviation.

All patients had a confirmed genetic diagnosis of Huntington’s disease (Table 1). One patient was pre-symptomatic and all other patients were symptomatic at various stages of the disease ranging from early through to moderately advanced stages. Patients had to be on a stable medication regime for at least four weeks prior to taking part in the study. Individuals were excluded from participation in the study if they had any other neurological conditions or a history of severe head injury, stroke or cerebral hemorrhages. To undergo brain imaging, participants also had to be eligible for MRI scanning i.e., without contraindications such as pacemakers, metal clips, stents, claustrophobia or significant chorea which would have prevented them from lying still in the scanner.

### Cognitive Assessment

Different aspects of working memory and executive functions, i.e., multi-tasking, working memory span, attention switching, distractor suppression and processing speed capacities were tested with the following standard paper and pencil neuropsychological tests: (1) The ability to multi-task was assessed with a dual task requiring the simultaneous crossing out of boxes on a sheet of paper and repeating sequences of digits at an individual’s short-term memory span (Baddeley, 1996). Participants’ performance was also assessed in each task individually. (2) The ability to switch attention was assessed with a verbal version of the trails test (VT), which requires the generation of letter and digit sequences in alternate order (Baddeley, 1996). Switching costs were assessed relative to generating letter or digit sequences alone. (3) The ability to suppress distracting and response incongruent information was measured with the Stroop task, which requires the reading of color words at baseline and filtering out incongruent ink color as interference condition (Trenerry et al., 1989). (4) Visuo-motor working memory capacities and processing speed were assessed with the Digit Symbol Substitution Test (DSST) from the Wechsler Adult Intelligence Scale (Wechsler, 1999). (5) Verbal and category fluencies were assessed using the letter cues “F”, “A”, “S” and “M”, “C”, “R” as well as the categories of “animals” and “boys’ names” and “supermarket items” and “girls’ names” respectively (Delis et al., 2001).

Previous pilot research identified correctly generated responses and response times in these tests as the most sensitive performance estimates (compared to error scores) in patients with HD (Metzler-Baddeley et al., 2014). Therefore the present study adopted these performance scores, leading to a total of 14 cognitive measurements, which are summarized in Table 5.

### MRI data acquisition

MRI data were acquired on a 3 T General Electric HDx MRI system (GE Medical Systems, Milwaukee) using an eight channel receiver only head RF coil at the Cardiff University Brain Research Imaging Centre (CUBRIC). The MRI protocol was comprised of T_1_-weighted, diffusion-weighted and quantitative magnetization transfer-weighted imaging. A T_1_-weighted anatomical fast spoiled gradient recalled (FSPGR) scan was acquired with the following parameters: 256 × 256 acquisition matrix, TR = 7.8 ms, TE = 2.9 ms, flip angle = 20, 172 slices, 1-mm slice thickness, FOV = 23 cm. Diffusion data were acquired employing a spin-echo echo-planar High Angular Resolution Diffusion Imaging (HARDI) (Tuch et al., 2002) sequence with diffusion encoded along 60 isotropically distributed orientations and six non-diffusion weighted scans according to an optimized gradient vector scheme (Jones et al., 1999) (96 × 96 acquisition matrix, TR/TE = 87 ms, *b*-value = 1200 s/mm^2^, 60 slices, 2.4-mm slice thickness, spatial resolution 1.8 × 1.8 × 2.4 mm). Diffusion data acquisition was peripherally gated to the cardiac cycle with a total acquisition time of ∼30 min depending on the heart rate.

An optimized 3D MT-weighted fast spoiled gradient recalled-echo (SPGR) sequence (Cercignani and Alexander, 2006) was used to obtain magnetization transfer-weighted data with the following parameters: TR/TE = 25.82/2.18 ms; Gaussian MT pulses, duration t = 14.6 ms; acquisition matrix = 96x96x60; BW = ± 244 Hz; FOV = 24 cm. The following off-resonance irradiation frequencies (Θ) and their corresponding saturation pulse amplitude (ΔSAT) for the 11 MT-weighted images were optimized using Cramer-Rao lower bound optimization [56]: Θ = [1000 Hz, 1000 Hz, 12,062 Hz, 47,185 Hz, 56,363 Hz, 2751 Hz, 1000 Hz, 1000 Hz, 2768 Hz, 2791 Hz, 2887 Hz] and their corresponding ΔSAT = [332°, 333°, 628°, 628°, 332°, 628°, 628°, 628°, 628°, 628°, 628°]. The longitudinal relaxation rate of the system was estimated by acquiring T_1_-maps using 3D SPGRs (TR = 6.85 ms, TE = 1.2 ms, FOV and resolution is the same as the MT sequence) with three different flip angles (theta = 15,7,3). Data for computing field (B_0_) maps were collected using two 3D spoiled, gradient recalled acquisitions (SPGR) with different echo-times (TE = 9 ms and 7 ms respectively; TR = 20 ms; matrix = 128 × 128; FOV = 220 mm; slice thickness 3 mm) (Jezzard and Balaban, 1995).

### MRI data processing

The diffusion-weighted HARDI data were corrected for distortions induced by the diffusion-weighted gradients, artifacts due to head motion and due to EPI-induced geometrical distortions by registering each image volume to the high-resolution T_1_–weighted anatomical images (Irfanoglu et al., 2012) with appropriate reorientation of the encoding vectors (Leemans and Jones, 2009) in ExploreDTI (Version 4.8.3) (Leemans et al., 2009). Since HD is associated with significant atrophy of brain tissue, DT-MRI metrics were corrected for cerebrospinal fluid based partial volume artifacts with the two compartment Free Water Elimination (FWE) approach (Pasternak et al., 2009) to derive maps of free water corrected FA, AD and RD (Pierpaoli and Basser, 1996).

MT-weighted SPGR volumes for each participant were co-registered to the MT-volume with the most contrast using an affine (12 degrees of freedom, mutual information) registration to correct for inter-scan motion using Elastix (normalized mutual information cost function) (Klein et al., 2010). The 11 MT-weighted SPGR images and T_1_-maps were modeled by the two pool Ramani’s pulsed MT approximation (Ramani et al., 2002). This approximation provided maps of the macromolecular proton fraction MMPF, which were nonlinearly warped to the T_1_-weighted SGPR image using the MT-volume with the most contrast as a reference using Elastix.

### Tractography

Whole brain tractography was performed for each participant in their native space using the damped Richardson-Lucy algorithm (dRL) (Dell'acqua et al., 2010), which (in contrast to diffusion tensor based tractography) allows the recovery of multiple fiber orientations within each voxels including those affected by partial volume. To reconstruct fiber tracts, dRL fiber orientation density functions (fODFs) were estimated at the center of each image voxel. Seed points were positioned at the vertices of a 2 × 2 × 2-mm grid superimposed over the image. The dRL tracking algorithm interpolated local fODF estimates at each seed point and then propagated 0.5 mm along orientations of each fODF lobe above a threshold peak of 0.05. This procedure allowed four potential streamlines to emanate from each seed point. Individual streamlines were subsequently propagated by interpolating the fODF at their new location and propagating 0.5 mm along the minimally subtending fODF peak. This process was repeated until the minimally subtending peak magnitude fell below 0.05 or the change of direction between successive 0.5-mm steps exceeded an angle of 45°. Tracking was then repeated in the opposite direction from the initial seed point. Streamlines whose lengths were outside a range of 10 mm to 500 mm were discarded.

Three dimensional fiber reconstructions of the WM tracts of interest were made by applying waypoint region of interest (ROI) gates (“AND”, “OR” and “NOT” gates following Boolean logic) to isolate specific tracts from the whole brain tractography data. ROIs were drawn manually by one operator (JBT) blind to the identity of each dataset on color-coded fiber orientation maps in native space guided by anatomical landmark protocols. Three distinct sections of the corpus callosum (CC) were reconstructed following the protocol by Hofer and Frahm (2006). These were sections maintaining fibers between the prefrontal cortices (CC1), the premotor and SMA cortices (CC2) and the motor cortices (CC3) (Fig.1A). The reconstructions of the anterior thalamic radiation (ATR) (Fig.1B) and the corticospinal tract (CST) (Fig.1D) were based on the anatomical fiber atlas by Wakana et al. (2004). The segmentation of fibers between the PFC and the caudate (Fig.1C) were guided by Kamali et al. (2010) and those between SMA and putamen (Fig.1E) by Leh et al. (2007).

### Extraction of subcortical BG and thalamic volume from T_1_-weighted anatomical images

Gray-matter subcortical volumes for left and right caudate, putamen, pallidum and thalamus were extracted from the individual T_1_-weighted images using the FMRIB Software Library (FSL) FIRST registration and segmentation tool (Patenaude et al., 2011) (www.fsl.fmrib.ox.ac.uk/fsl/fslwiki/FIRST). The FIRST procedure involves as a first step the registration of each individual’s T_1_-weighted image to the Montreal Neurological Institute (MNI) standard template with affine registration. During this step voxels outside subcortical regions are excluded using an MNI subcortical mask. FIRST then applies a Bayesian model of shape recognition to perform segmentation of subcortical structures. The segmented images were uploaded onto the original T_1_-weighted images and were visually inspected for correct registration for all participants. Quantitative volume measures from the BG and thalamus segmentations were extracted using the FSL statistics tool.

In addition all FIRST subcortical volumes were corrected for head size with the volumetric scaling factor derived from SIENAX version 2.5 (part of FSL4.1, http://www.fmrib.ox.ac.uk/fsl) (Smith et al., 2001). This involved skull extraction with BET and an affine-registration to the Montreal Neurological Institute (MNI) standard template. The volumetric scaling factor/value was extracted and used as the normalization factor to obtain for head size corrected volumes (i.e. by multiplying the volumetric scaling value with the subcortical volumes obtained from FIRST).

### Statistical analysis

All statistical analyses were carried out using SPSS Version 20 (IBM, 2011). All data were inspected for outliers defined as values larger or smaller than three times the standard deviation from the mean.

Since microstructural MRI metrics have been previously reported to be highly correlated between WM pathways (Penke et al., 2010, Penke et al., 2012) it is important to recognize that the 44 WM metrics [4 indices (MMPF, FA, AD, RD) x 11 tracts (CC1, CC2, CC3 and CST, ATR, PFC-caudate, SMA-putamen on both hemispheres)] may not be independent but could be impure measures of overlapping latent constructs. To account for this possibility we employed an exploratory principal component analysis (PCA) to identify the minimum number of uncorrelated principal components that explained together the maximum amount of variance in the microstructural data (Joliffe, 1986). PCA was also employed to reduce the dimensionality of the eight gray matter volume data (caudate, putamen, pallidum and thalamus on both hemispheres) and the 14 cognitive outcome measures (Table 5).

Due to the relatively small sample size for PCA (*n* = 32 for WM microstructural and gray matter volume data and *n* = 33 for cognitive data) we followed recommendations to limit the number of extracted components to a minimum (Preacher and MacCallum, 2002, de Winter et al., 2009). Selecting the number of components for data summary is always a trade-off between choosing too few components that may miss important underlying structures and too many components that reflect noise. Since there is no single recommended method we adopted the following approach: Firstly, we employed the SPSS default of the Kaiser criterion of including all components with an eigenvalue >1, secondly we inspected Cattell’s scree plots (Cattell, 1952) to identify the minimal number of components that accounted for most of the data variability and thirdly we assessed the selected components with regard to their interpretability. We used a PCA procedure with orthogonal Varimax rotation of the factor matrix, whereby each component has a small number of large loadings and a large number of small loadings. Table 3, Table 4, Table 5 summarize the component loadings for the WM microstructural, gray matter volumetric and cognitive data respectively. Per convention, loadings that exceeded a value of 0.5 were considered as significant.

We then tested for group differences in the principal component scores with independent *t*-tests and assessed the effect sizes with Cohen’s *d* (Cohen, 1988). Group differences in component scores were interpreted by referring to the variables with significant component loadings as highlighted in bold in Table 3, Table 4, Table 5.

Spearman’s rho correlation coefficients were calculated between WM microstructural component scores, gray matter volumetric component scores, cognitive component scores and the clinical measures from the UHDRS to assess disease-related brain-function relationships. Significant correlations were further assessed with partial correlations to control for potentially mediating variables of age, number of CAG repeats and years of education (YoE).

All statistical tests were corrected for multiple comparison errors with the Bonferroni correction with a family-wise alpha level of 5% (two-tailed) leading to a corrected *p*-value of <0.0167 for three independent t-tests for WM microstructure, *p* < 0.025 for two independent *t*-tests for cognition, *p* < 0.049 for one gray matter volumetric component and *p* < 0.003 for sixteen Spearman correlation coefficients.

## Results

Four individuals (HD11, HD12, HD21 and one control) turned out not eligible for MRI scanning due to claustrophobia and metal contamination and only performed the behavioral part of the study. One patient’s MRI data had to be discarded due to excessive motion (HD07). One control individual was excluded due to an incidental MRI finding. Thus, the analysis of the MRI data was based on 21 patients and 11 controls. Please also note that four patients at more advanced stages (HD07, HD10, HD12, HD13) could not perform all cognitive tasks. One patient’s (HD10) diffusivity data and one control participant’s BG volume data were identified as outliers. Results will be reported before and after outlier exclusion.

Table 2 summarizes information about demographic variables and performance in the Montreal Cognitive Assessment (MoCA) (Nasreddine et al., 2005) and in the revised National Adult Reading Test (NART-R) (Nelson, 1991) for those patients and controls whose MRI data were included in the analyses. Both groups were matched for age, sex and years of education but the patient group performed less well than the control group in the MoCA and the NART.

**Table 2:** Demographics and general cognitive profile of patients and controls

*Mean (SD)*	Patients (*n* = 21)	Controls (*n* = 11)	*t*-statistic (*p*-value)
*% female*	48	50	–
*Age*	45.6 (12.7)	51.4 (14.4)	*t*(30) = 0.96 (0.35)
*Years of Education*	13.33 (2.68)	15.0 (4.4)	*t*(30) = 1.32 (0.195)
*NART-IQ*	103 (12.9)	118.9 (7.5)	*t*(29)* = 3.7 (0.001)
*MoCA*	22.7 (5.6)	27.18 (1.7)	*t*(30) = 2.6 (0.015)

*Abbreviations:* MoCA = Montreal Cognitive Assessment score out of 30; NART-IQ = verbal IQ estimate based on the National Adult Reading Test.

* One patient could not perform the NART.

### Group differences in WM microstructure

With PCA, three components were extracted that explained 66% of the variability in the WM microstructural data (Table 3). The first component loaded positively on AD of left and right ATR, PFC-caudate fibers, SMA-putamen fibers, CST and the CC1, positively on RD of left and right ATR and PFC-caudate fibers and CC1, CC2 and CC3 and negatively on FA of CC1, CC2, CC3 and positively on FA of left SMA-putamen fibers. Since this component predominantly loaded highly on AD and RD it will be summarized as “diffusivity” component. The second component loaded highly on MMPF in all tracts but not on any DT-MRI indices and will hence be referred to as “MMPF” component. The third component loaded positively on FA of left and right ATR, PFC-caudate fibers, CC1 and right SMA-putamen fibers and negatively on RD of left and right SMA-putamen and CST. This component will be referred to as “FA” component.

**Table 3:** Rotated component matrix of the principal component analysis of the white matter microstructural data (*N* = 32)^a^

Tract-specific white matter microstructural metrics		“Diffusivity” component	“MMPF” component	“FA” component
*Macromolecular Proton Fraction (MMPF)*				
ATR	Left	−0.18	**0.77**	−0.03
	Right	−0.13	**0.87**	−0.17
CC1		−0.18	**0.94**	−0.12
CC2		−0.38	**0.86**	−0.06
CC3		−0.36	**0.86**	−0.01
PFC-caudate	Left	−0.26	**0.76**	0.31
	Right	−0.06	**0.83**	0.10
SMA-putamen	Left	0.01	**0.75**	0.15
	Right	−0.01	**0.82**	−0.04
CST	Left	0.01	**0.91**	−0.07
	Right	−0.13	**0.90**	−0.03

*Axial Diffusivity (AD)*				
ATR	Left	**0.86**	−0.21	0.24
	Right	**0.87**	−0.13	0.26
CC1		**0.72**	−0.15	0.37
CC2		0.44	0.19	−0.10
CC3		0.16	0.30	−0.24
PFC-caudate	Left	**0.73**	−0.13	0.28
	Right	**0.77**	−0.11	0.33
SMA-putamen	Left	**0.81**	−0.23	0.17
	Right	**0.76**	−0.21	0.16
CST	Left	**0.80**	−0.15	0.03
	Right	**0.71**	−0.42	−0.11

*Radial Diffusivity (RD)*				
ATR	Left	**0.79**	−0.09	−0.49
	Right	**0.74**	−0.10	−0.34
CC1		**0.91**	−0.13	−0.17
CC2		**0.91**	−0.04	−0.24
CC3		**0.88**	−0.02	−0.14
PFC-caudate	Left	**0.78**	−0.21	−0.36
	Right	**0.61**	−0.39	−0.44
SMA-putamen	Left	0.01	0.10	**−0.69**
	Right	0.01	0.03	**−0.72**
CST	Left	0.06	−0.10	**−0.52**
	Right	0.07	0.16	**−0.55**

*Fractional Anisotropy (FA)*				
ATR	Left	−0.07	−0.14	**0.86**
	Right	−0.02	−0.01	**0.59**
CC1		**−0.61**	0.07	**0.51**
CC2		**−0.84**	0.10	0.30
CC3		**−0.86**	0.14	0.11
PFC-caudate	Left	−0.16	0.13	**0.67**
	Right	−0.11	0.26	**0.77**
SMA-putamen	Left	**0.53**	−0.16	**0.49**
	Right	0.47	−0.13	**0.59**
CST	Left	0.41	0.06	0.35
	Right	0.43	−0.42	0.44

Loadings >0.5 are highlighted in bold. *Abbreviations:* ATR = Anterior Thalamic Radiation, CC = Corpus Callosum, CST = Cortico-spinal Tract, FA = Fractional Anisotropy, MMPF = Macromolecular Proton Fraction, PFC = Prefrontal Cortex, SMA = Supplementary Motor Area, RD = Radial Diffusivity.

a Rotation method: Varimax with Kaiser normalization.

**Table 4:** Component matrix of the principal component analysis of the subcortical gray matter volumetric data (*N* = 32)^a^

*Subcortical volume*	“BG volume” component	
*Caudate*	*Left*	**0.844**
	*Right*	**0.902**
*Putamen*	*Left*	**0.923**
	*Right*	**0.906**
*Pallidum*	*Left*	**0.833**
	*Right*	**0.868**
*Thalamus*	*Left*	**0.844**
	*Right*	**0.849**

Loadings >0.5 are highlighted in bold.

**Table 5:** Rotated component matrix of the principal component analysis of the cognitive data (*N* = 33)^a^

Cognitive Scores	“Executive function” component	“Verbal Span” component
*Single digit span (attempted)*	−0.013	**0.952**
*Single digit span (correct)*	0.077	**0.936**
*Dual task (attempted)*	0.143	**0.935**
*Dual task (correct)*	0.193	**0.922**
*Single box crossing*	**0.839**	0.171
*Dual box crossing*	**0.834**	0.218
*Stroop baseline RT*	**−0.791**	−0.182
*Stroop interference score*	**0.856**	0.021
*DSST*	**0.818**	0.254
*Verbal Trails baseline RT (*sec*)*	**−0.557**	−0.285
*Verbal trails switching RT (*sec*)*	**−0.873**	−0.203
*Verbal trails switching RT cost (*sec*)*	**−0.874**	0.163
*Verbal fluency*	**0.852**	0.049
*Category fluency*	**0.829**	0.225

Loadings >0.5 are highlighted in bold. DSST = Digit Symbol Substitution Test, RT = Reaction Time.

a Rotation method: Varimax with Kaiser normalization.

**Fig. 2:**
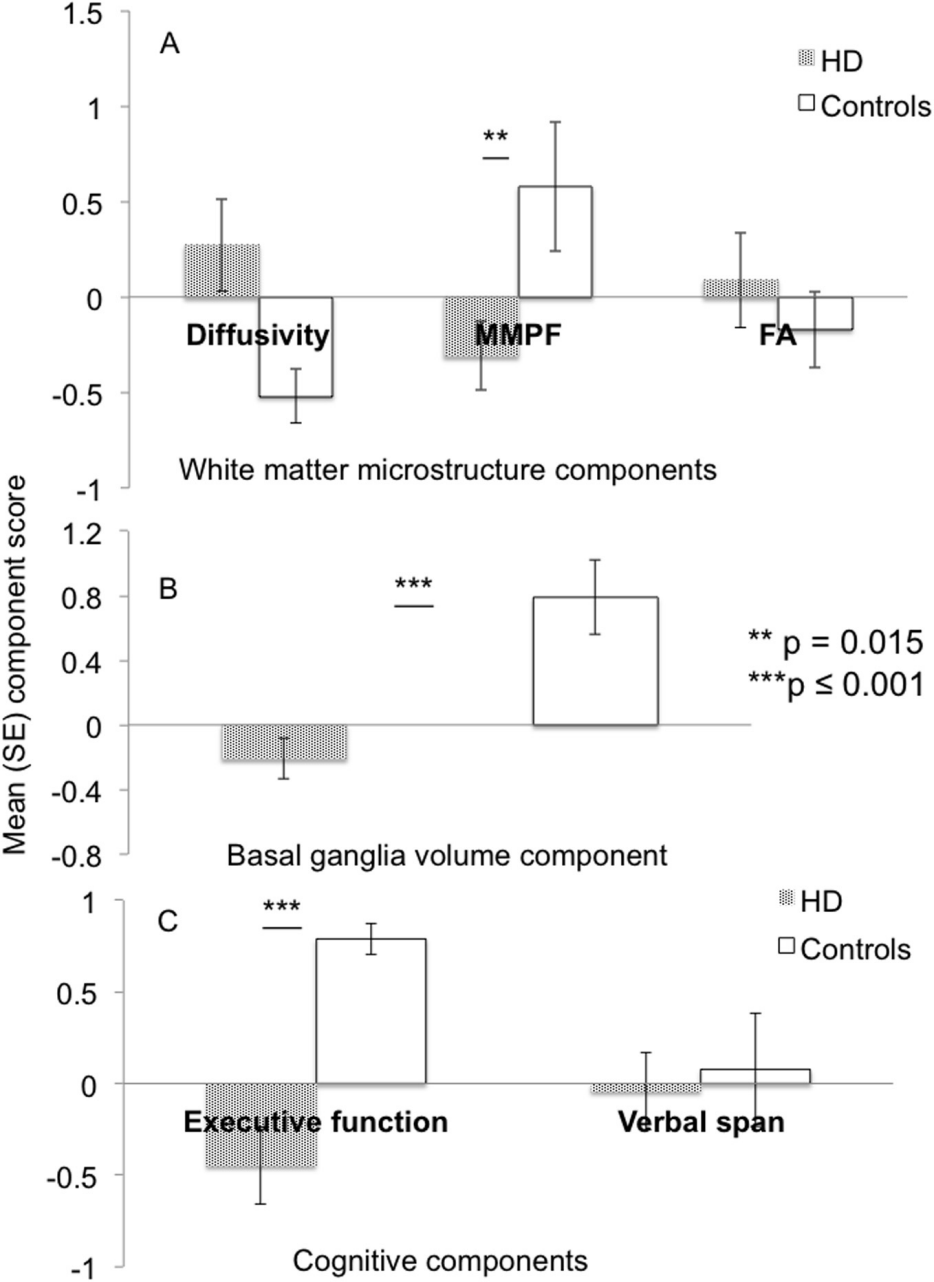
Group differences in white matter microstructure, basal ganglia volume and cognition. (A) Mean component scores for patients with Huntington’s disease (patterned bars) and controls (white bars) of the “diffusivity” component with positive loadings on axial and radial diffusivity, of the component with positive loadings on the macromolecular proton fraction (“MMPF component”) and the component with positive loadings on fractional anisotropy (“FA” component). Patients showed significantly reduced scores in the MMPF component, a trend for increased scores in the diffusivity component but no difference in the FA component. (B) Mean component scores of the “basal ganglia (BG) volume” component with positive loadings on volumes of the caudate, putamen, pallidum and the thalamus on both hemispheres. After the exclusion of one control participant’s outlier score HD patients showed significantly reduced BG volume relative to controls. (C) Mean component scores of the ‘executive function’ component with loadings on performance in the Stroop task, Verbal and Category Fluency, Verbal Trails, Digit Symbol Substitution task and box crossing under single and dual task conditions and the “verbal span” component with loadings on performance in digit span only. Patients showed significantly reduced executive function performance but did not differ in verbal working memory capacity from the controls. Abbreviations: HD = Huntington’s Disease, FA = Fractional Anisotropy, MMPF = Macromolecular Proton Fraction, SE = Standard error.

Independent *t*-tests revealed a significant group difference for the MMPF component [*t*(30) = 2.6, p = 0.015, *d* = 0.92] and a trend for the diffusivity component [*t*(30) = 2.2, p = 0.033, *d* = 0.94] [after outlier exclusion *t*(29) = 2.3, *p* = 0.028] but no difference for the FA component [*t*(30) = 0.69, *p* = 0.5, *d* = 0.25] (Fig.2A).

### Group differences in gray matter volume

One principal component for the subcortical volumetric data was extracted which explained 76% of the variation in the data and loaded highly on volumes of the caudate, putamen, pallidum and thalamus in both hemispheres (“BG volume” component). For the complete dataset there was no difference between the groups in this component [*t*(30) = 1.67, *p* = 0.104, *d* = 0.56]. However, after the exclusion of one control participant with an outlier BG volume component score, HD patients showed significantly reduced BG volume compared to healthy controls [*t*(29) = 4.15, *p* < 0.001] (Fig.2B).

### Group differences in cognition

The following two components that explained 76% of the variation in the cognitive data were extracted: the first component loaded on all executive function tasks i.e. performance in the Stroop, DSST, verbal trails, verbal and category fluency and box crossing performance under single and dual task condition and will therefore be described as “executive function” component. The second component loaded on performance scores in the digit span tasks only and will hence be labeled “verbal span” component. Patients differed from the controls in the executive function component [*t*(31) = 4.3, *p* ≤ 0.001, *d* = 1.7] but not in the verbal span component [*t*(31) = 0.33, *p* = 75, *d* = 0.1] (Fig.2c).

### Spearman’s rho correlations between WM microstructural components, BG volume component, cognitive components and clinical UHDRS measures

**Fig. 3:**
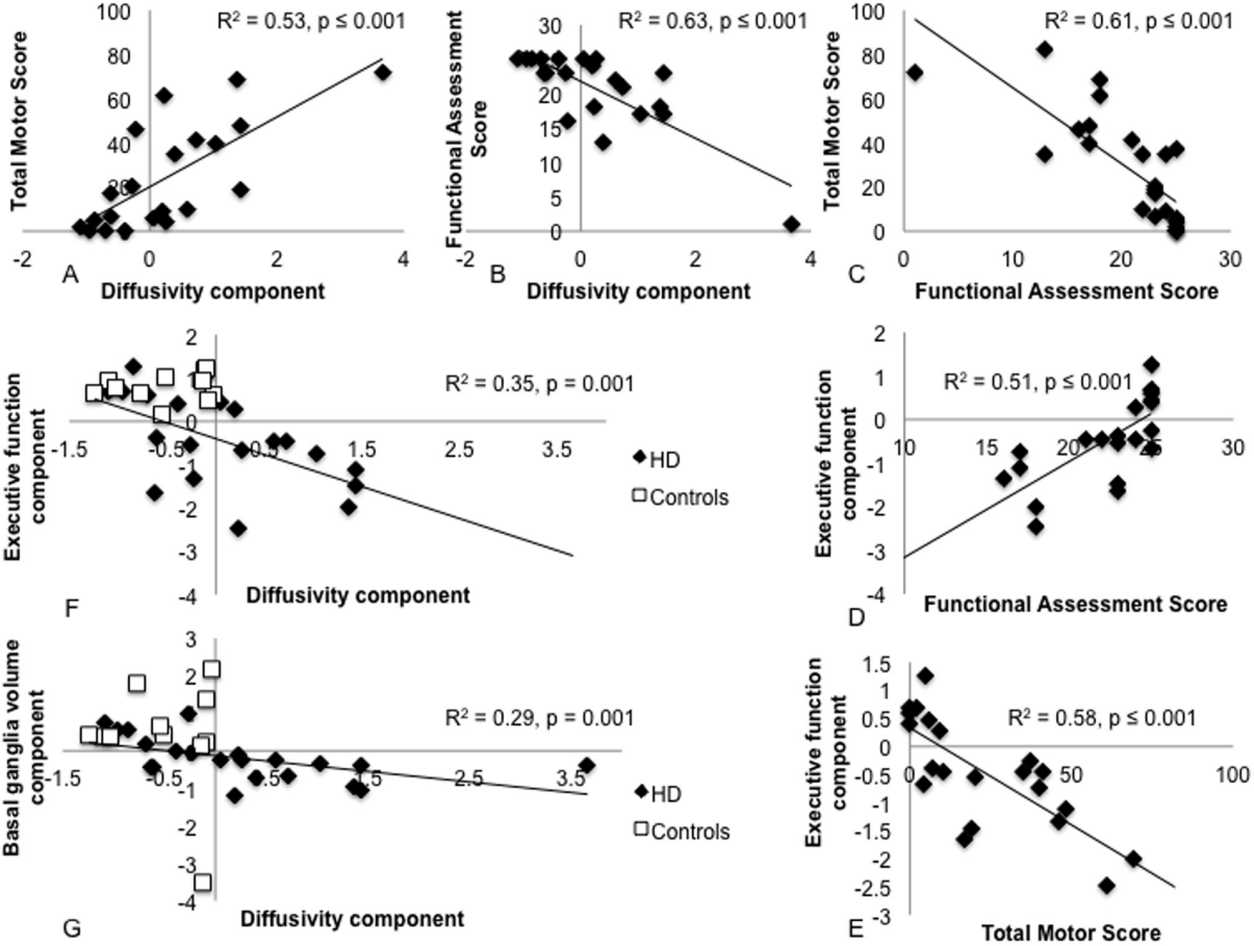
Spearman rho correlations between white matter microstructure, basal ganglia volume, cognition and clinical measures. (A) A positive correlation between patients’ inter-individual variation in the diffusivity component scores and their Total Motor Score of the United Huntington’s disease Rating Scale (UHDRS). Larger scores reflect more impaired performance or microstructure respectively. Please note that this correlation remained significant after the exclusion of one outlier that can be seen in the top right corner. (B) A negative correlation between patients’ inter-individual variation in the diffusivity component and their Functional Assessment Score (FAS) of the UHDRS. Lower FAS scores reflect more severe impairment. Please note that this correlation remained significant after the exclusion of the outlier in the left bottom corner. (C) A negative correlation between patients’ variation in the TMS and FAS which remained significant after outlier exclusion. (D) A positive correlation between patients’ FAS and their executive function component. (E) A negative correlation between patients TMS and their executive function component. (F) A negative correlations between inter-individual variation in the diffusivity and executive function component across patients (black diamond) and controls (white square). (G) A negative correlations between inter-individual variation in the diffusivity and basal ganglia volume component across patients (black diamond) and controls (white square).

There were significant correlations between the patients’ inter-individual variation in the diffusivity component and their performance in the two clinical measures of the UHDRS. A positive correlation was found with performance variation in the TMS (*r* = 0.74, *p* < 0.001, *n* = 21) (Fig.3A) and a negative correlation with performance variation in the FAS (*r* = −0.69, *p* < 0.001, *n* = 21) (Fig.3B). These correlations remained significant after the exclusion of the patient outlier that can be seen in Fig. 3 (TMS: *r* = 0.7, *p* = 0.001, *n* = 20; FAS: *r* = −0.65, *p* = 0.002, *n* = 20) but both were reduced when potential mediating effects of age, CAG repeats and YoE were partialled out (TMS: *r* = 0.67, *p* = 0.01; FAS: *r* = −0.55, *p* = 0.05).

Patients’ inter-individual variation in FAS correlated negatively with inter-individual differences in TMS (*r* = −0.85, *p* < 0.001) (Fig.3C). This correlation survived partialling out of age, CAG repeats and YoE (*r* = −0.91, *p* < 0.001) and the exclusion of one outlier (*r* = −0.83, *p* < 0.001). In addition, patients’ inter-individual differences in the executive component correlated positively with FAS scores (*r* = 0.79, *p* < 0.001, *n* = 21) (Fig.3D) and negatively with TMS scores (*r* = −0.78, *p* < 0.001, *n* = 21) (Fig.3E). These correlations remained significant after partialling out of age, CAG repeats and YoE (executive -TMS: *r* = −0.80, *p* < 0.001; executive -FAS: *r* = 0.75, *p* = 0.003).

For all participants, i.e., patients and controls, a negative correlation was present between inter-individual differences in the diffusivity component and the executive function component (*r* = −0.59, *p* = 0.001, *n* = 30) (Fig.3F) which remained significant after controlling for age and YoE (*r* = −0.62, *p* = 0.023). Finally, inter-individual differences in the diffusivity component also correlated negatively with the BG volume component (*r* = −0.61, *p* < 0.001, *n* = 32) (Fig.3G). This correlation remained significant after the exclusion of two outliers (one patient, one control) (*r* = −0.63, *p* < 0.001, *n* = 30) and after partialling out age and YoE (*r* = −0.65, *p* < 0.001). No other correlations were significant after multiple comparison correction (see Table 6).

**Table 6:** Correlations between brain structural component, cognitive components and clinical markers

*Spearman rho correlation coefficient (p-value)*	**“Diffusivity” component**	**“MMPF” component**	**“FA” component**	**“Basal ganglia” component**
***Total Motor Score***	**0.74 (<0.001)**	0.32 (0.15)	−0.03 (0.89)	*−0.52 (0.02*)
***Functional Assessment Score***	**−0.69 (<0.001)**	−0.24 (0.28)	0.10 (0.66)	*0.53 (0.01)*
***Executive function component***	**−0.59 (0.001)**	0.18 (0.35)	−0.34 (0.06)	*0.47 (0.01)*
***Verbal Span component***	−0.07 (0.71)	−0.09 (0.64)	0.12 (0.54)	−0.04 (0.84)

Correlations coefficients that were significant after Bonferroni correction are highlighted in bold. Trends defined as correlations significant at the uncorrected level are highlighted in italics.

## Discussion

Based on i. evidence indicating that myelin breakdown underpins WM damage in HD (Bartzokis et al., 2007, Ernst et al., 2014, Huang et al., 2015, Teo et al., 2016) and ii. histology evidence demonstrating that MMPF is highly sensitive to the myelin content of WM (Schmierer et al., 2007, Ou et al., 2009, Liu et al., 2015), the aim of this study was to investigate whether MMPF was a sensitive *in vivo* measure of WM degeneration in HD. Consistent with this hypothesis we observed a significant reduction in a component that loaded highly on MMPF in WM for HD patients relative to healthy controls (Fig.2A). The MMPF component loaded highly on all reconstructed WM pathways (Table 3) suggesting that myelin breakdown was present in all fiber bundles. Although MMPF is sensitive to WM myelin, it is important to recognize that this metric can also be influenced by changes in glia cells and water content due to neuroinflammation (Henkelman et al., 2001, Serres et al., 2009a, Serres et al., 2009b, Vavasour et al., 2011). In manifest HD it is likely that inflammation goes hand in hand with myelin breakdown (Silajdžić et al., 2013, Rocha et al., 2016). However, a recent CSF biomarker study found no evidence of neuroinflammation in premanifest HD (Vinther-Jensen et al., 2016). It would therefore be informative to study differences in MMPF in a cohort of premanifest gene-expansion carriers. This might help with teasing apart different biophysical contributions to MMPF and would allow to find out if myelin breakdown constitutes an early feature of HD pathogenesis that may precede other neurodegenerative processes in HD (Bartzokis et al., 2007). The MMPF component score of the one presymptomatic individual (HD03) included in the present study was −0.567 which differed more that three times the standard deviation from the control mean component score (*M* = 0.578, SD = 0.37). This observation suggests that MMPF might already be reduced prior to disease onset and that it would be worthwhile to investigate MMPF as an early disease biomarker in a group of asymptomatic gene carriers (Bartzokis et al., 2007). The novel contribution of the current study lies in the demonstration of MMPF reductions in manifest HD and in highlighting the potential of quantitative MRI markers in helping to better understand HD pathogenesis.

This study also replicated a number of findings that have previously been reported in the HD literature (e.g. Gerard et al., 2015; Poudel et al., 2014, Odish et al., 2015). Firstly, after the exclusion of one control outlier we found evidence of BG volume loss in patients relative to controls. BG atrophy is a well-established clinical feature of HD and striatal volume loss has been proposed as biomarker for clinical trials (Aylward, 2014). We also observed a trend for increases in the diffusivity component with positive loadings on AD and RD. This group comparison did not survive multiple-comparison correction but was of a comparable effect size (*d* = 0.94) as the MMPF effect (*d* = 0.92). Increases in AD and RD may arise due to multiple pathological changes likely to co-occur in manifest HD including demyelination, inflammation, axonal loss due to Wallerian degeneration and tissue atrophy, all of which will contribute to the manifestation of clinical symptoms. Indeed, we found strong correlations between patients’ inter-individual variations in the diffusivity component and variation in the BG volume component as well as performance variation in the UHDRS-TMS and FAS and the executive functioning component. These correlations were all in the expected directions with positive associations between diffusivity and TMS and negative associations with BG volume, FAS and executive function (Fig. 3). However, the correlations between the diffusivity component and the clinical measures of disease stage (TMS and FAS) were partially accounted for by patients’ differences in age, the number of CAG repeat lengths and YoE suggesting that DT-MRI diffusivities may be more general markers of disease stage rather than indicating specific alterations in WM microstructural properties. In contrast, FA was not sensitive to HD related differences in WM (Odish et al., 2015). If there are changes both in AD and RD then FA will remain constant and hence will not provide a sensitive marker for disease-related changes in WM microstructure (Acosta-Cabronero and Nestor, 2014). We propose that we did not find any correlations between clinical markers and the MMPF component because the latter may provide a more specific metric of myelin impairment that on its own may not be directly related to disease stages.

With regard to the cognitive and clinical measures we observed that patients’ TMS and FAS ratings were closely related with each other and with the executive function component, because patients at more advanced stages of disease showed poorer executive functioning. Although we found that HD patients relative to controls were significantly impaired in the executive functioning component they did not differ from controls in their verbal working memory span component. This dissociation suggests that the well-established impairments in multi-tasking and executive functioning in HD (Papoutsi et al., 2014, Mörkl et al., 2016, Stout et al., 2016) arise due to deficits in the control and manipulation of information processing rather than due to a reduced capacity for storing verbal information in working memory (Baddeley, 1996). An understanding of the nature of cognitive deficits associated with HD may provide useful guidance for future research into the efficacy of cognitive training and rehabilitation approaches in HD (Andrews et al., 2015)**.**

Our results were based on a relatively small sample size of patients and warrant replication in larger samples sizes. Future prospective longitudinal studies in presymptomatic mutation gene carriers far and close to disease onset are required to assess the utility of MMPF as a marker of early disease development and progression over time. In addition there was also some variation in patients’ medication and although we are not aware of any documented effects of these drugs on the here reported MRI indices we cannot completely rule out that there might have been any.

In summary, we provide novel *in vivo* evidence for reductions in MMPF, a proxy MRI marker of axon myelin, in human Huntington’s disease. Expanding on evidence from pathology and animal studies our results suggest that myelin breakdown may contribute to WM microstructural changes. Future studies in presymptomatic mutation gene carriers are required to clarify whether MMPF may provide an early biomarker of myelin breakdown in HD.
